# Downregulation of vimentin expression increased drug resistance in ovarian cancer cells

**DOI:** 10.18632/oncotarget.9970

**Published:** 2016-06-13

**Authors:** Yi Huo, Zhiguo Zheng, Yuling Chen, Qingtao Wang, Zhenyu Zhang, Haiteng Deng

**Affiliations:** ^1^ MOE Key Laboratory of Bioinformatics, School of Life Sciences, Tsinghua University, Beijing, China; ^2^ Zhejiang Tumor Hospital, Hangzhou, China; ^3^ Beijing Chaoyang Hospital Affiliated to Capital Medical University, Beijing, China

**Keywords:** ovarian cancer cell line, drug-resistance, proteomics, vimentin, cisplatin

## Abstract

Cisplatin and other platinum-based drugs have been widely used in the treatment of ovarian cancer, but most patients acquire the drug resistance that greatly compromises the efficacy of drugs. Understanding the mechanism of drug resistance is important for finding new therapeutic approaches. In the present study, we found that the expression of vimentin was downregulated in drug-resistant ovarian cancer cell lines A2780-DR and HO-8910 as compared to their respective control cells. Overexpression of vimentin in A2780-DR cells markedly increased their sensitivity to cisplatin, whereas knockdown of vimentin in A2780, HO-8910-PM and HO-8910 cells increased the resistance to cisplatin, demonstrating that vimentin silencing enhanced cisplatin resistance in ovarian cancer cells. Quantitative proteomic analysis identified 95 differentially expressed proteins between the vimentin silenced A2780 cells (A2780-VIM-KN) and the control cells, in which downregulation of endocytic proteins and the upregulation of exocytotic proteins CHMP2B and PDZK1 were proposed to contribute the decreased cisplatin accumulation in vimentin knockdown cells. Silencing of vimentin induced upregulation of cancer stem cell markers and both A2780-DR and A2780-VIM-KN cells were more facile to form spheroids than control cells under serum-free culture condition. Our results also revealed that vimentin knockdown increased the 14-3-3 mediated retention of Cdc25C in the cytoplasm, leading to inactivation of Cdk1 and the prolonged G2 phase arrest that allowed the longer period of time for cells to repair cisplatin-damaged DNA. Taken together, we demonstrated that vimentin silencing enhanced cells' resistance to cisplatin via prolonged G2 arrest and increased exocytosis, suggesting that vimentin is a potential target for treatment of drug resistant ovarian cancer.

## INTRODUCTION

Ovarian cancer causes the majority death in women with gynecological cancer. Patients with ovarian cancer are normally treated with surgical resection followed by platinum/taxane-based chemotherapy. However, the acquisition of resistance to currently available drugs during therapy is frequently present in ovarian cancer patients that have greatly compromised the therapeutic efficacy [1–3]. Understanding of the molecular mechanisms underlying drug resistance is urgently needed to improve patients' survival rates and qualities of their lives. Platinum-based drug resistance in ovarian cancer has been attributed to many factors including intracellular cisplatin inactivation, reduced intracellular drug accumulation, increased DNA repair and defects in cell death signaling pathways [4]. Intracellular cisplatin strongly binds to GSH, methionine, metallothionein and other cysteine-rich proteins, which decrease the amount of active cisplatin in cells [5, 6]. Reduced intracellular drug accumulation is regulated by CTR1, ATP7A and ATP7B. The knockout of CTR1 which contributes to the influx of cisplatin significantly enhances the resistance to cisplatin compared to control cells [7]. Overexpression of ATP7A or ATP7B responsible for the efflux of cisplatin increases the resistance to cisplatin [8]. The binding of cisplatin and DNA leads to DNA cross-links and induces cell death. Increased repair of damaged DNA contributes to the drug resistance [9–12]. GADD45 and ERCC1 in NER pathway were found to be upregulated in drug resistant cells [11, 12]. Defects in cell death signaling pathways are also present in drug resistant cells. For example, AKT was phosphorylated by DNA-PK which inhibited cisplatin-mediated apoptosis [13].

Our previous study showed that vimentin was downregulated in drug resistant ovarian cancer cell line A2780-DR [14]. Vimentin, a member of the intermediate filament (IF) family, is ubiquitously expressed in mesenchymal cells. As a cytoskeleton protein, vimentin is known to support and anchor the organelles and maintain cellular integrity [15]. In the recent years, increased vimentin expression has been found in a wide range of epithelial cancers including gastrointestinal tumors, prostate cancer, breast cancer, CNS tumors, lung cancer, and malignant melanoma [16–21]. Vimentin has been regarded as a canonical marker of epithelial–mesenchymal transition (EMT) [22], a cellular reprogramming process in which epithelial cells get mesenchymal phenotype in shape and motility. Upregulation of vimentin in cancers positively correlates with increased tumor invasion, growth and poor prognosis [15]. However, relatively little is known about the effects of vimentin expression on drug resistance in ovarian cancer cells. In addition to our previous study showing that vimentin was downregulated in cisplatin-resistant cells, vimentin downregulation was also present in ovarian cancer cells with acquired resistance to two tubulin-targeting drugs, peloruside A and laulimalide [23], suggesting that vimentin silencing promotes drug resistance in ovarian cancer cells.

In the present work, vimentin downregulation was confirmed in cisplatin-resistant ovarian cancer cell lines A2780-DR and HO-8910 compared to their respective drug-sensitive controls. Further studies demonstrated that vimentin knockdown in A2780, HO-8910-PM and HO-8910 cells increased the drug resistance to cisplatin and the overexpression of vimentin in A2780-DR cells increased the sensitivities to cisplatin. Differentially expressed proteins between A2780-VIM-KN cells and the control cells were identified showing that vimentin knockdown increased the expression levels of exocytotic proteins. We revealed that silencing of vimentin induced the prolonged G2 phase arrest and the acquired stem cell-like phenotype. Our data proposed a new mechanism underlying vimentin-mediated cisplatin resistance and suggested that vimentin was a new therapeutic target for treating drug-resistant ovarian cancer.

## RESULTS

### Expression of vimentin was downregulated in drug-resistant ovarian cancer cells

**Figure 1:**
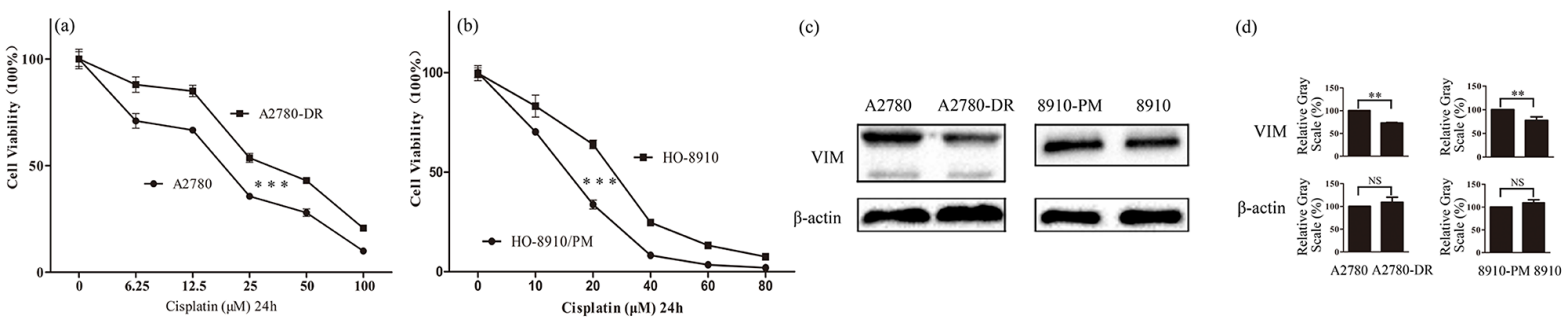
Correlation of vimentin expressions with cisplatin resistance in ovarian cancer cells **a.** Survival rates of A2780 and A2780-DR cells treated with different concentration of cisplatin for 24 h. **b.** Survival rates of HO-8910 and HO-8910-PM cells treated with different concentration of cisplatin for 24 h. **c.** Western blotting images of vimentin in A2780, A2780-DR, HO-8910 and HO-8910-PM cells. **d.** Gray scale analysis of western blotting images showing that the expression of vimentin was downregulated in cisplatin resistant cells.

In the present study, two pairs of cell lines, A2780/A2780-DR and HO-8910/HO-8910-PM were used to examine the effects of vimentin expression on drug resistance. In consistent with the previous report, A2780-DR displayed a higher resistance against cisplatin (Figure 1a). HO-8910 and HO-8910-PM cells were commonly used for studying metastasis in ovarian cancer, in which HO-8910-PM cells were derived from HO-8910 cells with a higher metastasis potential. The cell cytotoxicity assay revealed that HO-8910 cells displayed the higher resistance against cisplatin than HO-8910-PM cells (Figure 1b). The expression of vimentin was examined by western blotting showing that vimentin expression was downregulated in both drug resistant cells A2780-DR and HO-8910 (Figure 1c), in which vimentin expression was 1.5 fold lower in A2780-DR cells and 1.3 fold lower in HO-8910 cells than that in A2780 and HO-8910-PM cells, respectively, based on image gray scale analysis (Figure 1d). Downregulation of vimentin was also found in ovarian cancer cells resistant to peloruside A and laulimalide [23]. These results propose that the expression levels of vimentin are negatively correlated to drug resistance in ovarian cancer cells.

### Vimentin silencing increased cisplatin resistance in A2780 and HO-8910-PM cells

**Figure 2:**
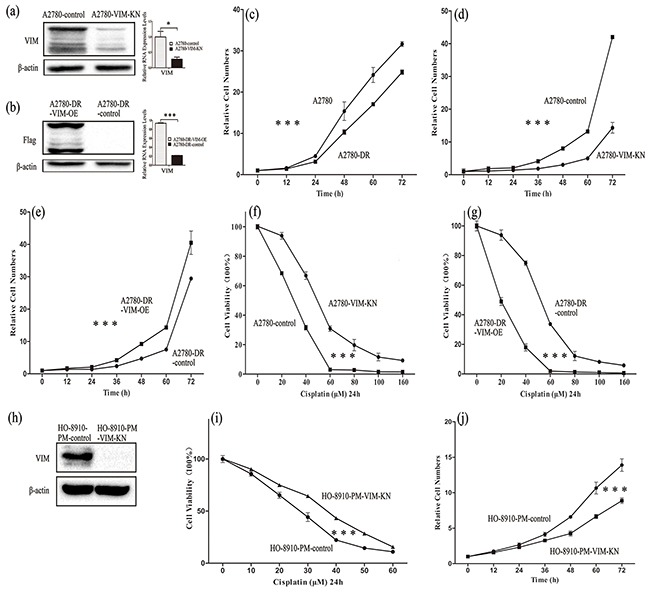
Characterization of A2780-VIM-KN, A2780-DR-VIM-OE, HO-8910-VIM-KN cells **a-b.** Western blotting and qPCR analysis confirming that the expression of vimentin was downregulated in A2780-VIM-KN cells and vimentin was overexpressed in A2780-DR-VIM-OE cells as compared to control cells. **c.** Growth curves of A2780 cells and A2780-DR cells showing that A2780-DR cells grew slower than A2780 cells. **d.** Growth curves of A2780-VIM-KN and control cells showing A2780-VIM-KN cells grew slower than control cells. **e.** Growth curves of A2780-DR-VIM-OE and control cells showing A2780-DR-VIM-OE cells grew faster than the control cells. **f–g.** Survival rates of A2780-VIM-KN, A2780-DR-VIM-OE and their control cells treated with different concentrations of cisplatin. **h.** Western blotting confirming that the expression of vimentin was downregulated in HO-8910-PM cells. **i.** Survival rates of HO-8910-PM and HO-8910-PM-VIM-KN cells treated with different concentration of cisplatin for 24 h. **j.** Growth curves of HO-8910-PM-VIM-KN and control cells showing HO-8910-PM-VIM-KN cells grew slower than control cells. All the results show the means of three independent experiments. Error bars indicate SEM. Data were analyzed using Student's t test. *p < 0.05, **p < 0.01 and ***p < 0.001.

To further explore effects of vimentin in drug-resistance, stable cell lines in which vimentin was silenced by vimentin-directed shRNA in A2780 (A2780-VIM-KN) or vimentin was overexpressed in A2780-DR cells (A2780-DR-VIM-OE) were established. The silencing and overexpression of vimentin in A2780 and A2780-DR cells were respectively verified by western blotting and qPCR analysis (Figure 2(a-b), Supplementary Figure S1(a-b)). The cell proliferation rates were measured by the CCK-8 assay showing that A2780 cells grew faster than A2780-DR cells in consistent with our earlier results (Figure 2c) whereas vimentin knockdown decreased the proliferation rate of A2780 cells (Figure 2d) and vimentin overexpression increased the proliferation rates of A2780-DR cells (Figure 2e). This demonstrated that the low expression of vimentin positively correlates with the low proliferation rates of ovarian cancer cells. The survival rates of A2780-VIM-KN, A2780-DR-VIM-OE and control cells were measured after cells were treated with different concentrations of cisplatin for 24h and results showed that silencing of vimentin in A2780 cells increased cell resistance to cisplatin (Figure 2f) whereas overexpression of vimentin in A2780-DR cells decreased cell resistance to cisplatin (Figure 2g). Similarly, downregulation of vimentin in HO-8910-PM and HO-8910 cells also increased cell resistance to cisplatin (Figure 2(h-i), Supplementary Figure S1(c-e)). Silencing of vimentin also decreased the proliferation rates of HO-8910-PM and HO-8910 cells (Figure 2j, Supplementary Figure S1f).

### Vimentin-silencing induced downregulation of proteins in cytoskeleton organization

**Figure 3:**
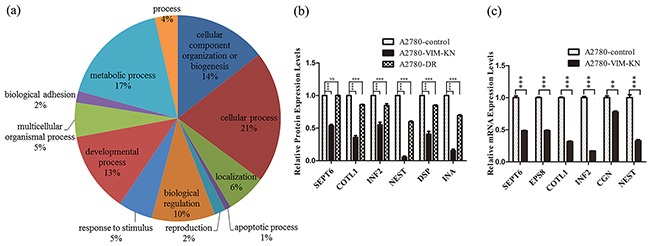
Analysis of differentially expressed proteins between A2780-VIM-KN and control cells **a.** Functional classification of differentially expressed proteins between A2780-VIM-KN and control cells with PANTHER (http://www.pantherdb.org). **b.** Graphical representation of expressions levels of selected proteins in A2780-DR, A2780-VIM-KN and control cells as determined by PRM analysis. **c.** Graphical representation of mRNA expressions levels of selected cell junction genes in A2780-VIM-KN and control cells. All the results show the means of three independent experiments. Error bars indicate SEM. Data were analyzed using Student's t test. *p < 0.05, **p < 0.01 and ***p < 0.001.

To understand the effects of vimentin on drug resistance in ovarian cancer cells, proteomic analysis was carried out to identify the differentially expressed proteins between A2780-VIM-KN and control cells. The experiments were carried out in three biological replicates, resulting in identification of 6362 proteins. Based on tandem mass tag (TMT) ratios (> 1.5 or <0.6) in proteins with more than two unique peptide matches, 95 differentially expressed proteins were found between A2780-VIM-KN and control cells, in which 47 proteins were upregulated and 48 downregulated (Supplementary Table S3 and S4). The Gene ontology (GO) analysis of the differentially expressed proteins was carried out and summarized according to their molecular functions via a pie plot by the PANTHER bioinformatics platform (http://www.pantherdb.org/) (Figure 3a). About half of differentially expressed proteins participated in the cellular processes and metabolic processes. Vimentin is an intermediate filament protein and its downregulation can induce cytoskeleton reorganization. Indeed, we found that proteins regulating the organization of the actin cytoskeleton were downregulated in A2780-VIM-KN cells including isoform I of septin-6 (SEPT6), epidermal growth factor receptor kinase substrate 8 (EPS8), coactosin-like protein (COTL1), isoform 2 of Inverted formin-2 (INF2), cofilin-2 (CFL2) and alpha-actinin-3 (ACTN3). The actin cytoskeleton strengthens the adherens junction which is formed primarily by cadherin [24, 25]. Intermediate filament proteins including alpha-internexin, nestin (NEST) and neurofilament heavy polypeptide were also down regulated in A2780-VIM-KN cells. Furthermore, desmoplakin (DSP) which plays a role in anchoring the intermediate filaments to the desmosomes [26, 27], and cingulin (CGN) that involved in regulation of the tight junction (TJ) [28] were downregulated in A2780-VIM-KN cells. Changes in protein expression levels of SEPT6, COTL1, INF2, NEST, DSP and INA between A2780-DR, A2780-VIM-KN and their respective control cells were confirmed by the parallel reaction monitoring (PRM)-based targeted mass spectrometry analysis (Figure 3b), indicating most differently expressed proteins between A2780-VIM-KN and control cells were also changed in the same trend between A2780 and A2780-DR cells. Furthermore, vimentin knockdown also induced downregulations in mRNA expression levels of *SEPT6, EPS8, CGN, COTL1, INF2* and *NEST* in A2780-VIM-KN cells as probed by qPCR analysis (Figure 3c). The qPCR analysis also confirmed changes in mRNA expressions of other proteins including FIGNL1, BDH2, DYNLT1, APBA2, LRP4, AGRN and STMN3 between A2780-VIM-KN and the control cells (Supplementary Figure S2).

### Vimentin silencing downregulated endocytosis and upregulated exocytosis leading to a decrease of cellular cisplatin accumulation

**Figure 4:**
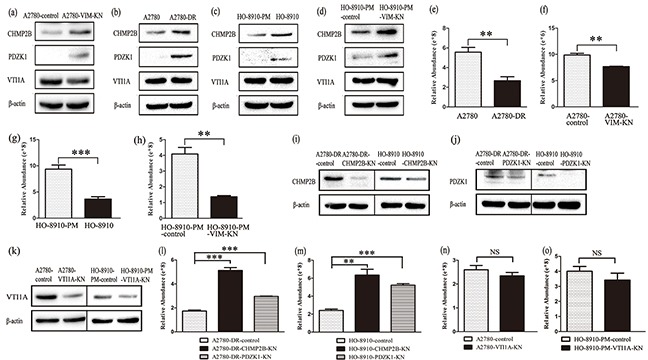
Vimentin silencing decreased the cellular cisplatin accumulation **a-d.** Western blotting images of the expression of CHMP2B, PDZK1 and VTI1A in (a) A2780-VIM-KN, (b) A2780-DR, (c) HO-8910, (d) HO-8910-PM-VIM-KN cells compared to their respective control cells. **e–h.** Graphical representation of accumulation of cisplatin in cisplatin treated (e) A2780-DR, (f) A2780-VIM-KN, (g) HO-8910, (h) HO-8910-PM-VIM-KN and their respective control cells. **i–k.** Western blotting analysis confirming that CHMP2B, PDZK1 and VTI1A were silenced in ovarian cancer cells. **l.** Graphical representation of the cellular cisplatin accumulation in cisplatin treated A2780-DR-CHMP2B-KN, A2780-DR-PDZK1-KN and control cells. **m.** Graphical representation of the cellular cisplatin accumulation in cisplatin treated HO-8910-CHMP2B-KN, HO-8910-PDZK1-KN and control cells. **n.** Graphical representation of the cellular cisplatin accumulation in cisplatin treated A2780-VTI1A-KN and control cells. **o.** Graphical representation of the cellular cisplatin accumulation in cisplatin treated HO-8910-PM-VTI1A-KN and control cells. All the results show the means of three independent experiments. Error bars indicate SEM. Data were analyzed using Student's t test. *p < 0.05, **p < 0.01 and ***p < 0.001.

Among differentially expressed proteins between A2780-control and A2780-VIM-KN cells, two proteins Na(+)/H(+) exchange regulatory cofactor NHE-RF3 (PDZK1) and charged multivesicular body protein 2b (CHMP2B) that regulated exocytosis were upregulated in A2780-VIM-KN cells, whereas isoform 1 of vesicle transport through interaction with t-SNAREs homolog 1A (VTI1A) involving in endocytosis was downregulated in A2780-VIM-KN cells. PDZK1 interacts with MRP2, a cellular cisplatin transporter and associated with multidrug resistance [29]. CHMP2B is the core component of endosomal secretary complex required for transport complex III (ESCRITIII) [30]. VTI1A, a member of SNARE protein family, promote endosome membrane fusion in endocytosis pathway [31, 32]. The expression levels of PDZK1 and CHMP2B were confirmed by qPCR analysis and western blotting while VTI1A was only slightly decreased in A278-VIM-KN cells (Figure 4a, Supplementary Figure S3(a-b)). CHMP2B and PDZK1 were also upregulated in A2780-DR, HO-8910 and HO-8910-PM-VIM-KN cells as compared to their respective control cells whereas changes in VTI1A expression were not significant among A2780-DR, HO-8910 and HO-8910-PM-VIM-KN cells (Figure 4(b-d), Supplementary Figure S3(c-e)), suggesting that CHMP2B and PDZK1 decreased cellular cisplatin accumulation in drug resistant cells.

To explore whether vimentin downregulation led to decreased intracellular cisplatin accumulation, the relative cellular cisplatin concentrations were measured in the drug resistant cells and control cells using mass spectrometry. Results revealed that the cisplatin concentration in A2780-DR, A2780-VIM-KN, HO-8910 and HO-8910-PM-VIM-KN cells were less than that in their respective control cells after cells were treated with 20 μM cisplatin for 24 h (Figure 4(e-h)), indicating that downregulation of vimentin decreased of cellular cisplatin accumulation to enhance cells' resistance to cisplatin.

To further validate that CHMP2B and PDZK1 upregulation decreased the cellular cisplatin accumulation, CHMP2B and PDZK1 were silenced in both A2780-DR and HO-8910 cells as verified by western blotting (Figure 4(i-j), Supplemental Figure S3f). Silencing of CHMP2B and PDZK1 in both A2780-DR and HO-8910 cells led to decreased cellular cisplatin accumulation (Figure 4(l-m)). On the other hand, silencing of VTI1A in both A2780 and HO-8910-PM cells had no effects on cellular cisplatin accumulation in these cells (Figure 4(k, n-o)). Our results declared that the vimentin downregulation increased CHMP2B and PDZK1 expression, which decreased the cellular cisplatin accumulation in drug resistant cells.

### A2780-VIM-KN and A2780-DR cells exhibited stem cell-like phenotypes

**Figure 5:**
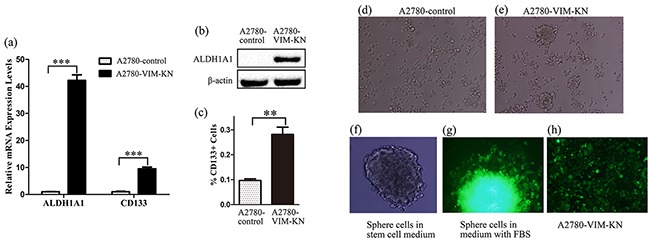
Vimentin knockdown induced a cancer stem cell-like phenotype in A2780 cells **a.** Graphical representation of mRNA expression levels of CD133 and ALDH1A1 in A2780-VIM-KN and control cells. **b.** Western blotting images of the stem cell marker ALDH1A1 in A2780-VIM-KN and control cells. **c.** FACS analysis of CD133 expression levels in A2780-VIM-KN and control cells. All the results show the means of three independent experiments. Error bars indicate SEM. Data were analyzed using Student's t test. *p < 0.05, **p < 0.01 and ***p < 0.001. **d.** The morphological image of A2780-control cells grown in ultralow adhesion tissue culture plates for 10 days. **e.** The morphological image of A2780-VIM-KN cells grown in ultralow adhesion tissue culture plates for 10 days. **f.** The morphological image of A2780-VIM-KN spheroids re-cultured in the stem cell medium. **g.** The morphological image of A2780-VIM-KN spheroid re-cultured in the normal medium. **h.** The morphological image of A2780-VIM-KN cells cultured in the normal medium.

Proteomics also showed that ALDH1A1 (Retinal dehydrogenase 1), a cancer stem cell marker, was upregulated in A2780-VIM-KN cells, as confirmed by western blotting and qPCR analysis (Figure 5(a-b), Supplementary Figure S4a). The other stem cell marker CD133 was also found to be upregulated in A2780-VIM-KN cells by qPCR analysis and flow cytometry analysis (FACS) (Figure 5(a, c)). To further validate that A2780-VIM-KN cells exhibited the characteristics of cancer stem cells, A2780-VIM-KN and control cells were planted into ultralow adhesion tissue culture plates. After 10 days, A2780-VIM-KN cells formed spheroid (Figure 5e) while control cells remained in its original morphology (Figure 5d). We re-cultured spheroids of cells in the stem cell medium and they remained in sphere shape after several generations (Figure 5f). However, the culturing of spheroids of cells in the medium with FBS resulted in the immediate adhesion of cells to the plate (Figure 5g) and restoration of cells' morphology of A2780-VIM-KN cells (Figure 5h). Similarly, ALDH1A1 and CD133 were also upregulated in A2780-DR cells as compared to A2780 cells (Supplementary Figure S4(b-d)), and consequently, A2780-DR cells were more facile to form spheroid than A2780 cells (Supplementary Figure S4e). CD133 was also upregulated in HO-8910-PM-VIM-KN cells as compared to the control cells (Supplementary Figure S4f). This suggests that vimentin knockdown reprograms cells to acquire cancer stem cell phenotype.

### Downregulation of vimentin led to a prolonged G2 arrest in drug-resistant cells

**Figure 6:**
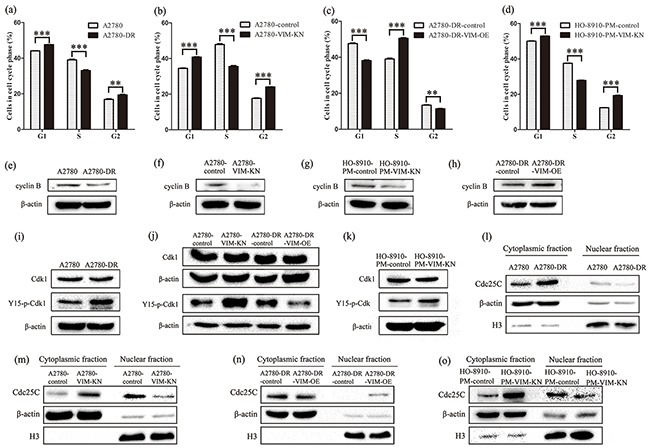
Vimentin silencing promoted a prolonged G2 arrest **a–d.** Cell cycle analysis of (a) A2780 and A2780-DR cells, (b) A2780-control and A2780-VIM-KN cells, (c) A2780-DR-control and A2780-DR-VIM-OE cell, and (d) HO-8910-PM-control and HO-8910-PM-VIM-KN cells. All the results show the means of three independent experiments. Error bars indicate SEM. Data were analyzed using Student's t test. *p < 0.05, **p < 0.01 and ***p < 0.001. **e–h.** Western blotting images of cyclin B expression in (e) A2780 and A2780-DR cells, (f) A2780-control and A2780-VIM-KN cells, (g) HO-8910-PM-control and HO-8910-PM-VIM-KN cells, (h) A2780-DR-control and A2780-DR-VIM-OE cell. **i–k.** Western blotting images of the expression of Cdk1 and phosphorylated Cdk1 at Y15 in (i) A2780 and A2780-DR cells, (j) A2780-control, A2780-VIM-KN cells, A2780-DR-control and A2780-DR-VIM-OE cell, (k) HO-8910-PM-control and HO-8910-PM-VIM-KN cells. **l–o.** Western blotting images of Cdc25C in cytoplasm and nucleus of (l) A2780 and A2780-DR cells, (m) A2780-control and A2780-VIM-KN cells, (n) A2780-DR-control and A2780-DR-VIM-OE cell, (o) HO-8910-PM-control and HO-8910-PM-VIM-KN cells.

The above results have shown that cells with higher cisplatin resistance tend to grow slower than the respective drug sensitive cells (Figure 2(c-e, j)); indicating that cell cycle progression varies among those different cells. Indeed, cell cycle analysis of A2780-DR, A2780-VIM-KN, A2780-DR-VIM-OE, and control cells showed that A2780-DR, A2780-VIM-KN and A2780-DR-control cells have higher G2/M phase accumulation than their drug sensitive counterparts (Figure 6(a-c), Supplementary S5a). Similar results were found in HO-8910-PM-VIM-KN and control cells (Figure 6d). These results suggested that drug resistant cells were prone to have a prolonged G2 phase. It has been known that cyclin B was highly expressed in M phase, and therefore, the low cyclin B expression was expected to be present in drug resistant cells. This was confirmed by western blotting analysis showing that cyclin B expression was lower in A2780-DR, A2780-VIM-KN, HO-8910-VIM-KN cells as compared to their respective control cells (Figure 6(e-g), Supplementary Figure S5(b-d)), whereas vimentin overexpression upregulated cyclin B in A2780-DR cells (Figure 6(h), Supplementary Figure S5e). The downregulation of cyclin B supported that drug resistant cells had prolonged G2 phase. Furthermore, no difference in Cdk1 expression that regulated G2-M transition was identified between A2780 and A2780-DR cells whereas phosphorylation of Cdk1 at Tyr15 (Y15) site was increased in A2780-DR cells (Figure 6i, Supplementary Figure S5f). Phosphorylation of Cdk1 was increased in A2780-VIM-KN cells and decreased in A2780-DR-VIM-OE cells as compared to control cells (Figure 6j, Supplementary Figure S5g). Similar results were found in HO-8910-PM-VIM-KN cells and control cells (Figure 6k, Supplemental Figure S5h). The phosphorylation of Y15 in Cdk1 by members of the Wee1/Mik1/Myt1 protein kinase family renders Cdk1 in the inactive state, resulting in a G2 arrest [33]. It is well known that activity of Cdk1 is regulated by Cdc25C. Expressions of Cdc25C in different subcellular localizations were examined by western blotting. The expression level of Cdc25C was higher in the cytoplasmic fraction but lower in the nuclear fraction of A2780-DR and A2780-VIM-KN cells as compared to the control cells (Figure 6(l-m), Supplementary Figure S5(i-j)). On the contrary, Cdc25C had the opposite cellular distribution in A2780-DR-VIM-OE compared to control cells (Figure 6n, Supplementary Figure S5k). Similar results were found in HO-8910-PM-VIM-KN and control cells (Figure 6o, Supplementary Figure S5l), indicating Cdc25C was retained in cytoplasm in drug resistant cells leading to the inactivation of Cdk1 and the prolonged G2 arrest.

## DISCUSSION

Downregulation of vimentin was identified in widely used drug resistant ovarian cancer cell lines in the present and previous studies [14, 23], suggesting that the low expression of vimentin correlated with drug resistance in ovarian cancer cells. Silencing of vimentin expression in A2780, HO-8910-PM and HO-8910 cells resulted in markedly increase the cell resistance to cisplatin further demonstrating that vimentin mediates drug resistance in ovarian cancer cells (Figure 2(f, i), Supplementary Figure S1f). This is rather interesting since vimentin is considered as a marker of mesenchymal cells and EMT is proposed to induce chemoresistance.

We carried out proteomic analysis to identify differentially expressed proteins between A2780-VIM-KN and control cells, showing that expressions of exocytotic proteins PDZK1 and CHMP2B were upregulated and that of endocytic protein VTI1A was downregulated, which contributed to the lower cisplatin accumulation in A2780-VIM-KN cells. This was further confirmed by the fact that overexpression of PDZK1 and CHMP2B in drug resistant cells decreased cellular cisplatin accumulation.

Downregulation of vimentin also decreased expressions of proteins participating in anchoring junction and tight junction in A2780-VIM-KN cells, which destabilized both anchoring junction and tight junction, leading to the emergence of cancer stem cell [34, 35]. This was confirmed by finding that cancer stem cell markers ALDH1A1 and CD133 were upregulated in A2780-DR and A2780-VIM-KN cells that were facile to form spheroids of cells under nutrient deprivation. CD133 was also upregulated in HO-8910-PM-VIM-KN cells compared with control cells (Supplementary Figure S4d). It is known that cancer stem cells exhibited the higher resistance to drug treatment [36, 37]. Therefore, we propose that vimentin-knockdown enhanced drug resistance in A2780 cells is partially arisen from the acquired cancer stem cell properties.

Our results also showed that downregulation of vimentin induced a prolonged G2 arrest in drug-resistant cells. The G2 arrest is triggered when cells experience DNA damage during G2 phase or sensitize unrepaired damage accumulated in prior S or G1 phases, which allows the longer period of time to repair the damaged DNA and to prevent segregation of damaged chromosomes [38–40]. G2 arrest is regulated by subcellular localization of Cdc25C. When Cdc25C is phosphorylated and remains in cytosol by binding to 14-3-3 proteins, Cdk1 is highly phosphorylated and in inactive state that induces G2-M cell cycle arrest [41–47]. Previous studies demonstrated that phosphorylated vimentin interacts with 14-3-3 protein to prevent the binding of 14-3-3 with other proteins [48]. Our results propose that downregulation of vimentin in cisplatin resistant cells increases availability of 14-3-3 proteins, which binds to Cdc25C and retains Cdc25C in the cytoplasm. This led to the inactivation of Cdk1 and the prolonged G2 arrest that allows sufficient time to repair cisplatin induced DNA damage and prevents cells proceed to necrosis or apoptosis [49].

Taken together, our results demonstrate that vimentin silencing in ovarian cancer cells upregulates proteins of the exocytotic process to decrease cellular cisplatin accumulation. Vimentin knockdown also reprograms cells to acquire cancer stem cell properties and induces a prolonged G2 arrest which contributes to drug resistance. Vimentin is a potential therapeutic target for treatment of resistance ovarian cancers.

## MATERIALS AND METHODS

### Chemicals and reagents

Dulbecco's modified Eagle's medium (DMEM), phosphate buffered saline (PBS), penicillin/streptomycin, fetal bovine serum were purchased from Wisent (Montreal, Canada). Dithiothreitol (DTT) was purchased from Merck (Whitehouse Station, NJ). Iodoacetamide (IAA) was purchased from Sigma (St Louis, MO). Sequencing grade modified trypsin was purchased from Promega (Fitchburg, WI). The TMT labeling kit was purchased from Thermo-Pierce Biotechnology (Rockford, IL). Anti-VIM antibody, anti-CHMP2B antibody, anti-PDZK1 antibody, anti-VTI1A antibody, anti-Cdk1 antibody, anti-Cdc25C antibody, anti-Y15-p-Cdk1 antibody and anti-H3 antibody were from Proteintech (Wuhan, China). Anti-ALDH1A1 antibody, anti-β-actin antibody and anti-flag antibody were from Sigma (St Louis, MO). Anti-CD133 antibody was from Miltenyi Biotec (Bergisch Gladbach, Germany).

### Cell culture

Human epithelial ovarian cancer cell lines A2780 and A2780-DR were grown in DMEM medium supplemented with 10% FBS and penicillin (100 U/ml)–streptomycin (100 μg/ml) at 37°C with 5% CO_2_ in a humidified incubator. Human epithelial ovarian cancer cell lines HO-8910 and HO-8910-PM were obtained from the cell bank of the Chinese Academy of Sciences (Shanghai, China) and cells were grown in DMEM medium supplemented with 10% FBS and 1% penicillin/streptomycin at 37°C in a humidified incubator with 5% CO_2_.

### Construction of vimentin-knockdown A2780, HO-8910-PM and HO-8910 cell line

The shRNA against vimentin was designed with an online tool (http://hannonlab.cshl.edu/GH_research.html). The sequence of vimentin targeting shRNA was AACACACTCAGTGCAGCAATAT. NCi was non-targeting scrambled control of shRNA. The oligonucleotides were annealed and inserted into the pll3.7 shRNA expression vector to generate shRNA. Pll3.7-VIMi or Pll3.7-Nci plasmids were transfected with packing vectors into 293T cells using PEI. 48 h later, the lentivirus particles in cell culture supernatant were then collected and concentrated with PEG6000. Then, precipitated lentivirus particles were resuspended in PBS and added into the A2780, HO-8910-PM and HO-8910 cells plated the day before transfection with 5 μg/mL polybrene. Cells expressing Pll3.7 plasmid co-expressed GFP. After 72 h, a single GFP-expressing cell was sorting into one single well in a 96-well plate by FACS. A2780-VIM-KN, HO-8910-PM-VIM-KN and HO-8910-VIM-KN cell lines which are with high effectiveness of inhibiting vimentin expression evaluated by western blotting and qPCR were selected and used in the present study and A2780, HO-8910-PM and HO-8910 cells transfected with lentivirus vectors containing non-targeting scrambled shRNA were used as control cells.

### Generation of A2780-DR cells stably overexpressing vimentin

Vimentin (GenBank accession number: 003380.3) was cloned from cDNA reversely transcripted from RNA of A2780-DR cells. The Flag-tag was inserted at the C terminus of vimentin and subcloned into pLVX-IRES-ZsGreen1 expression vector. Vimentin overexpression cells (A2780-DR-VIM-OE) were constructed and isolated using the protocol described above. A2780-DR cells transfected with empty vector were used as the control.

### Cell proliferation assay with CCK-8

Cells were seeded in 96-well plates with 2000 cells/well. Cell proliferation rate was determined with the Cell Counting Kit-8 (CCK-8) according to the manufacturer's instructions (Dojindo Laboratories, Kumamoto, Japan). Briefly, CCK-8 reagents were added into wells after cells grew for 0, 12, 24, 36, 48, 72, 84, 96 h respectively. Absorbance at 450 nm was measured 2 h after CCK-8 addition.

### Cell cytotoxicity assay

Cells (8 × 10^3^ each) were seeded in 96-wellplates and cultured for 16 h followed by cisplatin treatment at different concentrations (0, 20, 40, 60, 80 and 160 μM) in triplicates for 24 h. Cell numbers was assessed by measuring absorbance at 450 nm with the CCK-8 assay. Cell viability was calculated as the percentage of variable cells compared with untreated cells.

### Proteomics analysis

Proteomic analysis was carried out in biological triplicate. Briefly, A2780-VIM-KN and control cells were lysed with 8 M urea in PBS pH 7.4. Equal amount of proteins from each samples (200 μg) were reduced with 1 mM dithiotreitol for 1 h and alkylated with 5.5 mM iodoacetamide for 40 min in the dark. Proteins were digested with sequencing grade modified trypsin for 16 h at 37°C. The digestion was stopped with 10 % trifluoracetic acid, after which peptides were desalted using HLB extraction cartridges and eluted using 1 ml methanol. Extracts were then centrifuged in a speedvac to reduce the volume. After that, peptides were redissolved in 100 μl 200 mM Tetraethylammonium Bromide (TEAB) and labeled with TMT sixplex labeling reagent for 1 h at room temperature according to the manufacture's instruction. The reaction was then quenched by adding 5 μl of 5% hydroxylamine in TEAB and incubating 15 min. The TMT-labeled peptides were combined and desalted using C18 sep-pak cartridges. The elution extracts were centrifuged in a speedvac to reduce the volume. Then the TMT-labeled peptides complex was separated by HPLC. For HPLC separation, the TMT-labeled peptides were separated with a C-18 column (Xbridge^TM^BEH 300 C18 5 μm Waters) at a flow rate 1 ml/min by the following gradient elution. Mobile phase A was 100% H_2_O and mobile phase B consisted of 98% acetonitrile and 2% H_2_O. The pH of both mobile phase A and B were adjusted to 10. The fractions were centrifuged in a speedvac to reduce the volume and analyzed by LC-MS/MS.

For LC-MS/MS analysis, the TMT-labeled peptides were separated by a 120 min gradient elution with a flow rate of 0.250 μl/min in a Thermo-Dionex Ultimate 3000 HPLC system, which was directly connected with a Thermo Scientific Q Exactive mass spectrometer. The analytical column was a home-made C-18 (300 Å, 5 μm, Varian, Lexington, MA) resin packed fused silica capillary column (75 μm ID, 150 mm length; Upchurch, Oak Harbor, WA). Mobile phase A consisted of 0.1% formic acid, and mobile phase B consisted of 100% acetonitrile and 0.1% formic acid. The Q Exactive mass spectrometer was operated by Xcalibur 2.1.2 software in the data-dependent acquisition mode and 10 data-dependent MS/MS scans at 29% normalized collision energy followed a single full-scan mass spectrum in the orbitrap (400 −1800 m/z, 60,000 resolution).

The peak lists from LC-MS/MS analysis were generated with Proteome Discoverer software (version 1.4.1.14, release date of December, 2012). The MS/MS spectra from each LC-MS/MS run were searched against the *human.fasta* database downloaded from Uniprot (release date of January 10, 2015; 89105 sequences) using an in-house Sequest HT Algorithmin Proteome Discoverer software. Common contaminants were included in the database. The search criteria were the followings: full tryptic specificity was required; one missed cleavage was allowed; carbamidomethylation (C) and TMT sixplex (K and N-terminal) were set as the fixed modifications; the oxidation (M) was set as thevariable modification; precursor ion mass tolerance was set at 20 ppm for all MS acquired in an orbitrap mass analyzer; and the fragment mass tolerance was set at 20 mmu for all MS2 spectra acquired in Q Exactive mass spectrometer. Peptide spectral matches (PSM) were validated using the Percolator provided by Proteome Discoverer software based on q-values at a 1% false discovery rate (FDR). A peptide whose sequence is only assigned to a given protein group was considered as unique. The false discovery rate was also set to 0.01 for protein identifications. Relative protein quantification was performed using Proteome Discoverer software (Version 1.4) according to manufacturer's instructions on the reporter ion intensities per peptide. Proteins with at least two unique peptides were regarded as confident identifications and were further quantified. Protein ratios were calculated as the median of all peptide hits belonging to a protein. Quantitative precision was expressed as protein ratio variability. The mass spectrometry proteomics data have been deposited to the ProteomeXchange Consortium via the PRIDE partner repository with the data set identifier PXD002825. The files now can be accessed with the username ‘reviewer11713@ebi.ac.uk’ and password ‘igi3e9MG’, and they will be completely available upon the publication of this manuscript.

### Western blotting and qPCR

For western blotting analysis, equal amount of proteins of A2780-control or A2780-VIM-KN cells were denatured at 100°C boiled 5 min with SDS loading buffer. The proteins were transferred to PVDF transfer membrane by electroblotting after SDS-PAGE separation. Membranes were probed with the indicated antibodies overnight at 4°C followed by immunoblotting analysis. β-actin was internal control. The gray intensity analysis of western blotting images was carried out by imageJ software. Total RNA was reversely transcribed using HIScript 1^st^ Strand cDNA Synthesis Kit (Vazyme, Nanjing, China). The primers for quantitative RT-PCR of cDNA were listed in Supplementary Table S1.

### Validation of differentially expressed proteins by parallel reaction monitoring

Equal amount of proteins from A2780-control cells and A2780-VIM-KN cells (80 μg) were separated by 1D SDS-PAGE, respectively. The gel bands of interest were excised from the gel, reduced with 25 mM of DTT and alkylated with 55 mM iodoacetamide which was followed by in-gel digestion with sequencing grade modified trypsin at 37°C overnight. The peptides were extracted twice with 0.1% trifluoroacetic acid in 50% acetonitrile aqueous solution for 30 min and then dried in a speedvac. Peptides were redissolved in 20 μl 0.1% trifluoroacetic acid and 6 μl of extracted peptides were analyzed by Thermo Scientific Q Exactive mass spectrometer in parallel reaction monitoring (PRM) mode. The acquisition method contained a full scan MS and a PRM event. The PRM event targeted the precursor ion for each selected peptide in ±4 min monitoring windows depending on their elution time. The selection of target precursor ions was performed based on the normal proteomics data acquired in data dependent acquisition mode. Lists of target peptides, target precursor ions, and selected fragment ions are provided in Supplementary Table S2. PRM acquisition was performed using a resolution of 17,500, individual isolation windows of 2 h, target AGC values of 1 × 10^6^, and equal individual fill times within each subset with maximum values of 100 ms. Fragmentation was performed with a normalized collision energy of 25, and MS/MS scans were acquired with a starting mass of m/z 100. Data analyses were typically performed using Xcalibur (Thermo Scientific). Mass tolerance of fragment ions was set at 20 mmu. Fragment ions resulting from neutral losses and low m/z ions (typically below m/z 200–300) were omitted. At least three fragment ions with the highest intensity were selected to ensure accuracy. The peak areas of fragment ions were used to calculate the relative intensity of precursor ion for selected peptides. At least two peptides were selected for the quantification of one protein. The means of the relative intensity of selected peptides represented the relative expression level of proteins.

### Spheroid forming assays

1×10^6^ A2780-VIM-KN and control cells were seeded into 6-well ultralow adhesion tissue culture plates (Corning Incorporated, USA) in 2 ml DMEM with glutamine and penicillin/streptomycin. When spheroids were formed, cells were collected and grown in 60 mm cell culture plate in stem cell medium: serum free DMEM media supplemented with 10% serum replacement (Gibco), 10 ng/ml human recombinant epidermal growth factor (EGF) and 10 ng/ml basic fibroblast growth factor (bFGF). For the differentiation of spheroid cells, cells were grown in DMEM with 10% FBS and penicillin (1 U/ml)–streptomycin (100 μg/ml).

### Cell cycle analysis

Cells were harvested by trypsinization, washed 3 times by ice-cold PBS and fixed with 70% ethanol overnight at 4°C. The fixed cells were resuspended in PBS, treated with RNase and stained with PI followed by FACS analysis. The percentage of cells in each cell cycle phase was assessed using Modfit software.

### Cell cytoplasm and nucleus extraction

Cells were harvested by trypsinization, washed 3 times with ice-cold PBS and the packed cell volume (PCV) was estimated. The cell pellet was resuspended gently in 5 × PCV of lysis buffer (10 mM HEPES, pH 7.9, with 1.5 mM MgCl_2_, 10 mM KCl and 1 mM DTT) and incubated on ice for 15 minutes, allowing cells to swell. The suspended cells were centrifuged for 5 minutes at 420 × g and the pellet of packed cells was resuspended in 2 × PCV of lysis buffer. The cell suspension was drawn slowly into the syringe with a narrow-gauge and then ejected with a single rapid stroke. Repeated ten times to make sure cell lysis reached 80-90%, but the nucleus of lysed cells remained intact. The disrupted cells in suspension were centrifuged for 20 min at 10,000–11,000 × g and the supernatant was the cytoplasmic fraction. The crude nuclei pellet was washed 3 times by lysis buffer and resuspended in 2/3 × PCV of extraction buffer (20 mM HEPES, pH 7.9, with 1.5 mM MgCl_2_, 0.42 M NaCl, 0.2 mM EDTA, 25% (v/v) Glycerolc and, 1 mM DTT). The nucleus suspension was shaken gently for 30 min and centrifuged for 20 min at 10,000-11,000 × g and the supernatant was nuclear fraction.

### Experimental design and statistical rationale

All experiments were performed in biological triplicates. In one experiment, the forward and reverse labeling was used to test the reliability of the quantitation method. No significant differences were observed for proteins identified with two or more unique peptides and no other technique replicates were performed. Statistical analysis was carried out with GraphPad Prism 5.0 software on data related to cell proliferation assay, cell cytotoxicity assay, PRM analysis and qPCR. Significant differences in the data were determined by Student's t test. P values of < 0.05 were considered significant.

## SUPPLEMENTARY FIGURES AND TABLES










