# Supplementary material for: Downregulation of vimentin expression increased drug resistance in ovarian cancer cells
Source: Oncotarget. 2016 Jun 13;7(29):45876–88. doi: 10.18632/oncotarget.9970 (PMC5216767; doi:10.18632/oncotarget.9970)
Supplement: Supplementary file 1 [file oncotarget-07-45876-s001.pdf]

Downregulation of vimentin expression increased drug resistance in ovarian cancer cells

SUPPLEMENTARY FIGURES AND TABLES

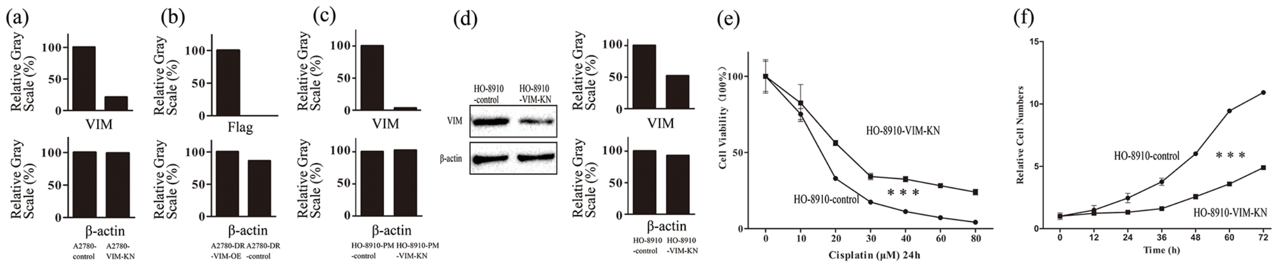

Supplementary Figure S1: a–c. Gray scale analysis of western blotting images. (a) Images presented in Figure 2a. (b) Images presented in Figure 2b. (c) Images presented in Figure 2h. **d.** Western blotting images and gray scale analysis confirming that vimentin was silenced in HO-8910-VIM-KN cells. **e.** Survival rates of HO-8910-VIM-KN and control cells treated with different concentrations of cisplatin. **g.** Cell growth curves of HO-8910-control and HO-8910-VIM-KN cells. All the results show the means of three independent experiments. Error bars indicate SEM. Data were analyzed using Student's t test. *$p < 0.05$,**$p < 0.01$ and *** $p < 0.001$.

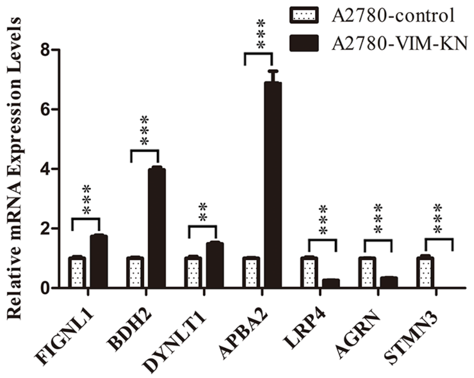

Supplementary Figure S2: Graphical representation of mRNA expression levels of FIGNL1, BDH2, DYNLT1, APBA2, LRP4, AGRN and STMN3 in A2780-VIM-KN and control cells. All the results show the means of three independent experiments. Error bars indicate SEM. Data were analyzed using Student's t test. *$p < 0.05$,**$p < 0.01$ and *** $p < 0.001$.

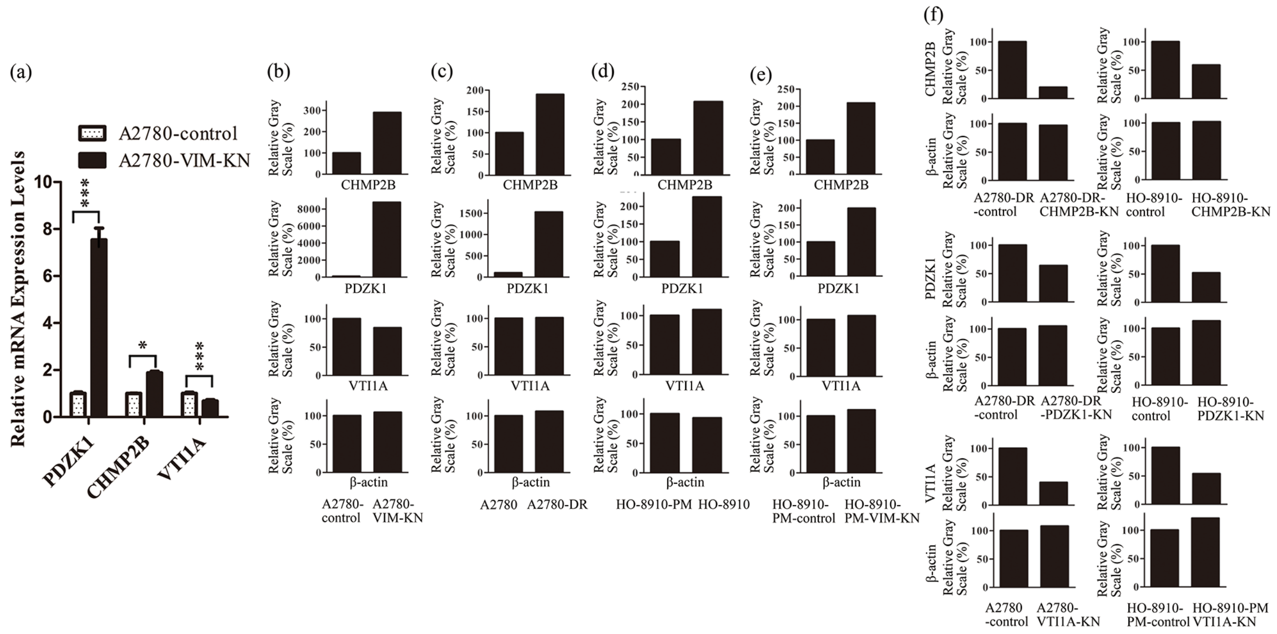

Supplementary Figure S3: a. Graphical representation of mRNA expressions levels of PDZK1, CHMP2B and VTI1A in A2780-VIM-KN and control cells. All the results show the means of three independent experiments. Error bars indicate SEM. Data were analyzed using Student's t test. $^{*}p < 0.05$, $^{**}p < 0.01$ and $^{***}p < 0.001$. **b–e.** Gray scale analysis of western blotting images. (b) Images presented in Figure 4a. (c) Images presented in Figure 4b. (d) Images presented in Figure 4c. (e) Images presented in Figure 4g. (f) Images presented in Figure 4(i-k).

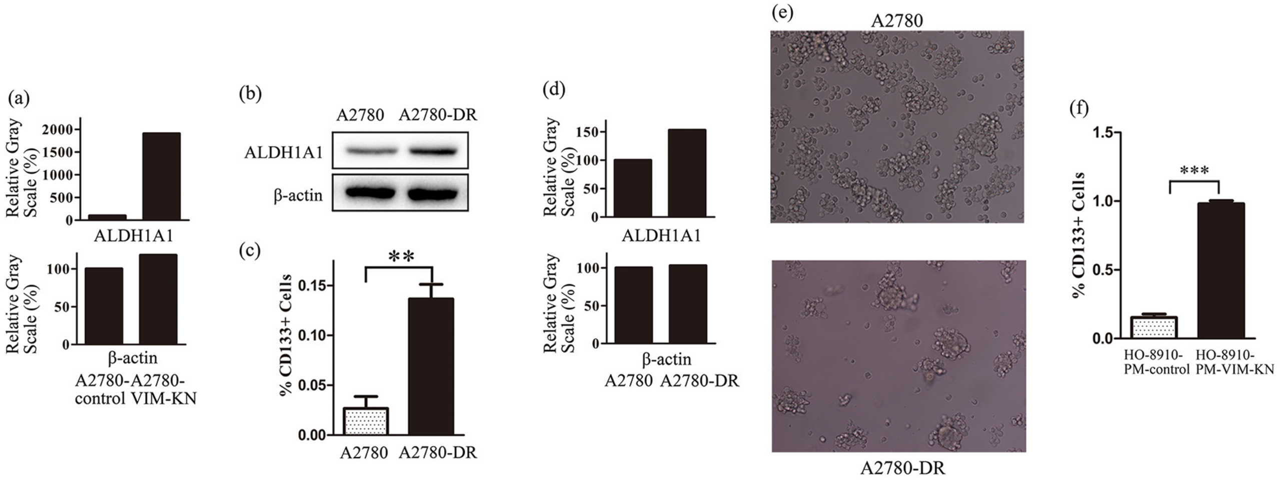

Supplementary Figure S4: a. Gray scale analysis of western blotting images presented in Figure 5b. **b.** Western blotting images of the stem cell marker ALDH1A1 in A2780-DR and control cells. **c.** FACS analysis of CD133 expression levels in A2780-DR and control cells. **d.** Gray scale analysis of western blotting images presented in Supplementary Figure S4b. **e.** FACS analysis of CD133 expression levels in HO-8910-PM-VIM-KN and control cells. All the results show the means of three independent experiments. Error bars indicate SEM. Data were analyzed using Student's t test. $^{*}p < 0.05$, $^{**}p < 0.01$ and $^{***}p < 0.001$.

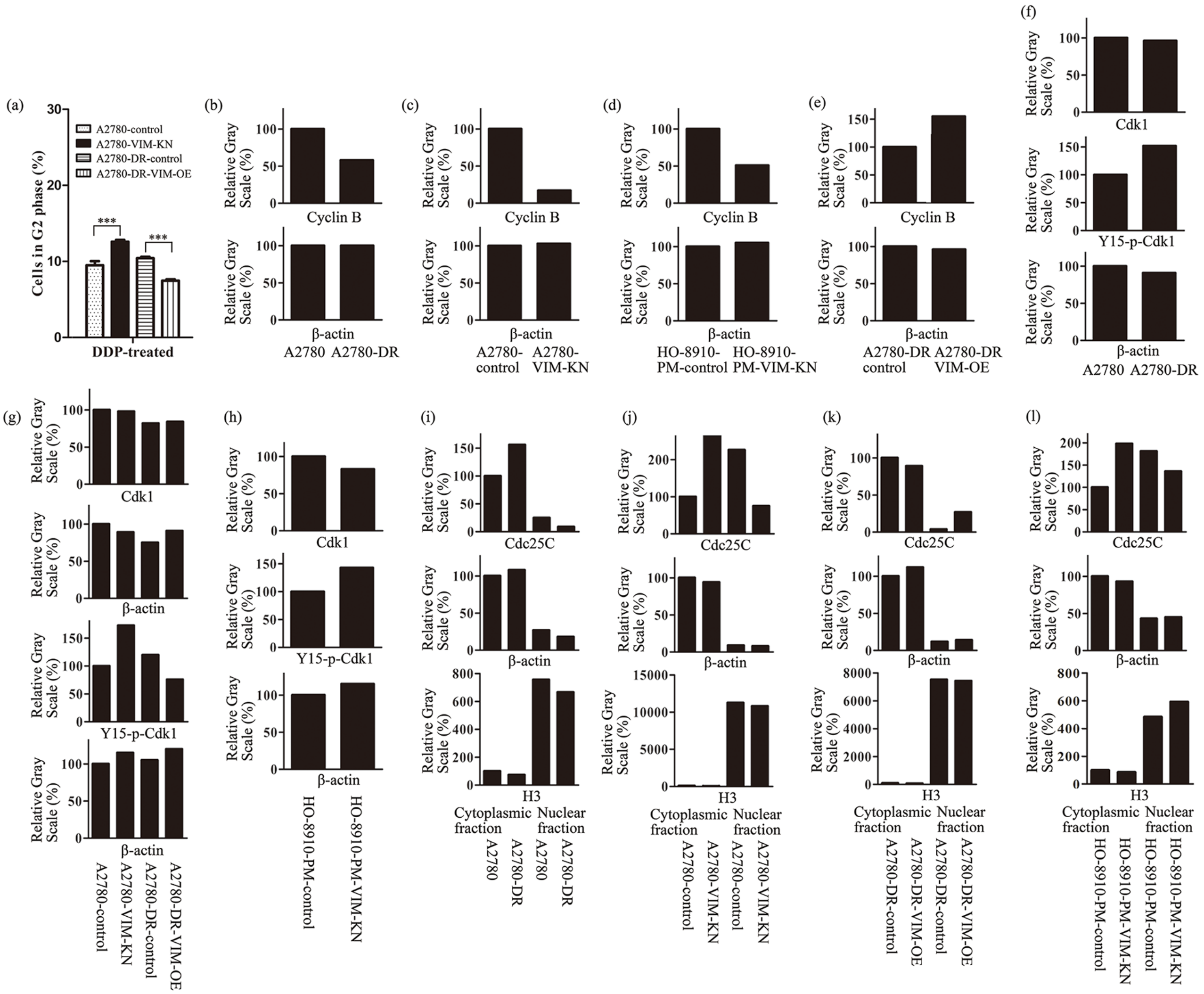

Supplementary Figure S5: a. Graphical representation of the percentage of cells in G2 phase in A2780-VIM-KN, A2780-DR-VIM-OE and control cells under cisplatin treatment. All the results show the means of three independent experiments. Error bars indicate SEM. Data were analyzed using Student's t test. *p<0.05, **p<0.01 and ***p<0.001. **b–j.** Gray scale analysis of western blotting images. (b) Images presented in Figure 6e. (c) Images presented in Figure 6f. (d) Images presented in Figure 6g. (e) Images presented in Figure 6h. (f) Images presented in Figure 6h. (g) Images presented in Figure 6j. (h) Images presented in Figure 6k. (i) Images presented in Figure 6l. (j) Images presented in Figure 6m. (k) Images presented in Figure 6n. (l) Images presented in Figure 6o.

Supplementary Table S1: Primers for qPCR analysis.

See Supplementary File 1

Supplementary Table S2: Proteins for PRM analysis.

See Supplementary File 2

Supplementary Table S3: Upregulated Proteins in A2780-VIM-KN cells.

See Supplementary File 3

Supplementary Table S4: Downregulated Proteins in A2780-VIM-KN cells.

See Supplementary File 4
