# Supplementary material for: Downregulation of vimentin expression increased drug resistance in ovarian cancer cells
Source: Oncotarget. 2016 Jun 13;7(29):45876–88. doi: 10.18632/oncotarget.9970 (PMC5216767; doi:10.18632/oncotarget.9970)
Supplement: Supplementary file 2 [file oncotarget-07-45876-s002.docx]

| Gene Name | | | Primer | |
| --- | --- | --- | --- | --- |
| VIM-F | | | GAACCTGAGGGAAACTAATC | |
| VIM-R | | | GAAAGGCACTTGAAAGCT | |
| PDZK1-F | | | AAAATCCCTATTGTTTCCTCCCTG | |
| PDZK1-R | | | AAGAATGTGAGGCTGTACTGTGGG | |
| VTI1A-F | | | CAAATAGCAGTGGAAACCGAGCAA | |
| VTI1A-R | | | TCTTCGCAACATCCCTGTCAGAAT | |
| CHMP2B-F | | | AGAAGAAAGCCAGGATATTGTGAA | |
| CHMP2B-R | | | TTGAAGTAGAGGCAGATGGTAAGC | |
| SEPT6-F | | | ACACCCTGTTCAACACCA | |
| SEPT6-R | | | AAGCCAACTGTGCTAACG | |
| EPS8-F | | | TGTGAGCCTGATTGATTT | |
| EPS8-R | | TGTTTCCCTCCTTTACTG | |  |
| COTL1-F | | CCTCATCCCACCTCCATC | |  |
| COTL1-R | | GAAATACTCCCTCCGTCAA | |  |
| INF2-F | | GAAGCGAAGGAAGAAGCG | |  |
| INF2-R | | TGGGTAAGGCACGGAGTTT | |  |
| CGN-F | | GGACATTTCAAGCCAACT | |  |
| CGN-R | | CCAGGGAGCACAATAACA | |  |
| NEST-F | | CAGAAACTCAAGCACCACT | |  |
| NEST-R | | TCCACCGTATCTTCCCAC | |  |
| FSCN1-F | | ACAGCGTCACCCGTAAGC | |  |
| FSCN1-R | | GGAACTCCAGCGTGTAGCC | |  |
| BDH2-F | | CAAGAAAGAATACAAGCCAGAGGA | |  |
| BDH2-R | | CAGGGTTACCAGTTACATAAGCAG | |  |
| DYNLT1-F | AAACTTTAAGCCAACTCACCAAGC | | | |
| DYNLT1-R | CAGGTCAAATAGACAGTCCGAAGG | | | |
| APBA2-F | CCAGGAAGGCAAGAAGCAGTATAAGA | | | |
| APBA2-R | TGAGAAGTGGATGAGGTCGTCGTT | | | |
| AGRN-F | TACGGCAACGAGTGTCAGC | | | |
| AGRN-R | TCACAGTCACGGAGGCAGAT | | | |
| LRP4-F | CCTCGGACTTCGTATGTG5 | | | |
| LRP4-R | AGCATCTTCCTTCCACCT | | | |
| STMN3-F | AAGCGGCAGCCACAAGTC | | | |
| STMN3-R | GGCGTCCAAGGGAAAGAA | | | |
| ACTIN-F | ACTTAGTTGCGTTACACCCTTTCTTG | | | |
| ACTIN-R | CTGTCACCTTCACCGTTCCAGTTT | | | |
