# Supplementary material for: Downregulation of vimentin expression increased drug resistance in ovarian cancer cells
Source: Oncotarget. 2016 Jun 13;7(29):45876–88. doi: 10.18632/oncotarget.9970 (PMC5216767; doi:10.18632/oncotarget.9970)
Supplement: Supplementary file 3 [file oncotarget-07-45876-s003.docx]

Supplemental Table 2 Proteins for PRM analysis

Target Protein Target Peptides RT Time (min)

Target Precursor Ions (m/z)

Selected Fragment Ions (m/z)

937.42596

479.26224

SEPT6

| TVPLAGHVGFDSLPDQLVNK | 63.55 | 703.04498 |
| --- | --- | --- |
| SVSQGFCFNILCVGETGLGK | 77.67 | 1087.0304 |
| YDGSTIVPGEQGAEYQHFIQQCTDDVR | 61.78 | 1038.46631 |
| SKFALITWIGENVSGLQR | 74.93 | 1010.05469 |
| EFVISDRKELEEDFIK | 56.14 | 666.34839 |

COTL1

SLDDEVNAFK 48.87 569.27991

822.3985

365.21902

817.44757

601.36938

954.00391

661.35303

920.45227

1033.5365

1189.52966

1140.9928

1239.06836

1359.70276

959.49054

1472.78699

811.42639

754.88324

860.95929

INF2

|  | | | 1216.63733 |
| --- | --- | --- | --- |
| SSQLLWEALESLVNR | 91.08 | 872.95667 | 1030.56006 |
|  |  |  | 901.51031 |
|  |  |  | 387.189 |
| ISDALLQLTCVSCVR | 67.49 | 868.42928 | 994.48907 |
|  |  |  | 881.39514 |
|  |  |  | 802.44226 |
| ALEEQNELLSAELGGLR | 66.99 | 921.4826 | 915.526 |
|  |  |  | 1028.61035 |
|  |  |  | 1110.57983 |
| GPPAPAPEVEELAR | 49.42 | 716.87811 | 1278.66882 |
|  |  |  | 716.39142 |
|  |  |  | 872.45984 |
| LELQQLQAER | 45.17 | 614.33887 | 744.40051 |
|  |  |  | 985.54279 |
|  |  |  | 1000.5868 |
| LLEAQIASGGVVDPVNSVFLPK | 77.06 | 1127.14709 | 1427.7893 |
|  |  |  | 1585.8596 |
|  |  |  | 965.5198 |
| NLPLADQGSSHHITVK | 35.15 | 572.97205 | 640.32372 |
|  |  |  | 745.39423 |
|  |  |  | 922.49432 |
| TIADLELHYQEFIR | 65.78 | 874.45575 | 885.43567 |
|  |  |  | 1105.58257 |

NEST

DSP

|  | | | | 944.47974 |
| --- | --- | --- | --- | --- |
|  | HSAEVAGYQDSIGQLENDLR | 58.77 | 734.68976 | 652.30542 |
|  |  |  |  | 759.40051 |
|  |  |  |  | 471.72507 |
| INA | HLREYQDLLNVK | 44.03 | 509.94882 | 528.26709 |
|  |  |  |  | 695.88739 |
|  |  |  |  | 909.44263 |
|  | RPPASDGLDLSQAAAR | 38.64 | 542.2851 | 603.32147 |
|  |  |  |  | 681.3316 |
