# Supplementary material for: Downregulation of vimentin expression increased drug resistance in ovarian cancer cells
Source: Oncotarget. 2016 Jun 13;7(29):45876–88. doi: 10.18632/oncotarget.9970 (PMC5216767; doi:10.18632/oncotarget.9970)
Supplement: Supplementary file 4 [file oncotarget-07-45876-s004.docx]

| Accession | Description | Score | Coverage (%) | Unique Peptides | PSMs | VIM-KN/  control | Ratio Variability [%] | MW [kDa] |
| --- | --- | --- | --- | --- | --- | --- | --- | --- |
| Q9BUT1 | 3-hydroxybutyrate dehydrogenase type 2 | 19 | 21 | 3 | 4 | 1.5 | 43 | 26.7 |
| P17643 | 5,6-dihydroxyindole-2-carboxylic acid oxidase | 5 | 5 | 3 | 3 | 3.0 | 34 | 60.7 |
| Q5STZ8 | ATP-binding cassette sub-family F member 1 (Fragment) | 53 | 37 | 3 | 20 | 1.5 | 9 | 38.3 |
| C0H5Y7 | BAK1 protein | 8 | 39 | 2 | 2 | 1.6 | 42 | 16.9 |
| P51911 | Calponin-1 | 52 | 49 | 10 | 17 | 2.2 | 37 | 33.1 |
| P49662 | Caspase-4 | 9 | 7 | 3 | 3 | 2.0 | 31 | 43.2 |
| Q9C0F1 | Centrosomal protein of 44 kDa | 20 | 17 | 4 | 5 | 1.7 | 27 | 44.1 |
| Q9UQN3 | Charged multivesicular body protein 2b | 24 | 29 | 8 | 8 | 1.7 | 29 | 23.9 |
| B5MCA1 | Coiled-coil domain-containing protein 104 | 10 | 11 | 2 | 3 | 1.5 | 18 | 36.1 |
| P05997 | Collagen alpha-2(V) chain | 34 | 7 | 8 | 11 | 2.0 | 19 | 144.8 |
| P29279 | Connective tissue growth factor | 91 | 63 | 19 | 29 | 2.2 | 33 | 38.1 |
| Q00534 | Cyclin-dependent kinase 6 | 16 | 15 | 3 | 6 | 2.0 | 7 | 36.9 |
| Q5VTU3 | Dynein light chain Tctex-type 1 | 13 | 37 | 2 | 3 | 1.6 | 24 | 10.2 |
| Q8IWV7 | E3 ubiquitin-protein ligase UBR1 | 16 | 4 | 3 | 4 | 1.5 | 23 | 200.1 |
| E7ER77 | Endoplasmic reticulum metallopeptidase 1 | 6 | 3 | 2 | 3 | 1.6 | 11 | 93.1 |
| P58107 | Epiplakin | 33 | 8 | 4 | 11 | 1.6 | 14 | 555.3 |
| Q5JTV1 | Glucocorticoid modulatory element binding protein 2, isoform CRA a | 10 | 9 | 2 | 3 | 1.6 | 39 | 51.2 |
| P09210 | Glutathione S-transferase A2 | 6 | 12 | 2 | 3 | 2.2 | 64 | 25.6 |

| Q5JSK8 | High mobility group nucleosome-binding domain-containing protein 5 (Fragment) | 32 | 73 | 7 | 12 | 3.0 | 81 | 11.4 |
| --- | --- | --- | --- | --- | --- | --- | --- | --- |
| Q9HBK9-2 | Isoform 2 of Arsenite methyltransferase | 18 | 23 | 5 | 5 | 1.6 | 16 | 31.1 |
| Q6PIW4-2 | Isoform 2 of Fidgetin-like protein 1 | 7 | 9 | 2 | 3 | 1.6 | 44 | 61.6 |
| P05121-2 | Isoform 2 of Plasminogen activator inhibitor 1 | 8 | 7 | 2 | 2 | 2.5 | 44 | 43.4 |
| O75157-2 | Isoform 2 of TSC22 domain family protein 2 | 21 | 5 | 2 | 7 | 2.0 | 48 | 76.3 |
| P14210-3 | Isoform 3 of Hepatocyte growth factor | 16 | 11 | 5 | 5 | 1.8 | 29 | 82.5 |
| Q0ZGT2-4 | Isoform 4 of Nexilin | 35 | 11 | 5 | 9 | 1.5 | 11 | 72.7 |
| Q08209-5 | Isoform 5 of Serine/threonine-protein phosphatase 2B catalytic subunit alpha isoform | 27 | 17 | 3 | 10 | 1.5 | 47 | 51.2 |
| O75369-8 | Isoform 8 of Filamin-B | 534 | 45 | 86 | 146 | 2.6 | 44 | 281.5 |
| Q13557-12 | Isoform Delta 12 of Calcium/calmodulin-dependent protein kinase type II subunit delta | 10 | 8 | 2 | 3 | 1.7 | 1 | 54.1 |
| P14618-2 | Isoform M1 of Pyruvate kinase PKM | 956 | 75 | 2 | 276 | 3.2 | 16 | 58.0 |
| P05787 | Keratin, type II cytoskeletal 8 | 429 | 18 | 6 | 188 | 2.0 | 11 | 53.7 |
| P30740 | Leukocyte elastase inhibitor | 67 | 42 | 14 | 18 | 1.7 | 12 | 42.7 |
| B7Z3X7 | Methyltransferase-like protein 16 | 8 | 10 | 2 | 2 | 1.8 | 88 | 38.9 |
| Q9Y605 | MORF4 family-associated protein 1 | 14 | 27 | 2 | 3 | 1.5 | 17 | 14.6 |
| P84022 | Mothers against decapentaplegic homolog 3 | 9 | 8 | 2 | 3 | 1.5 | 7 | 48.0 |
| P29966 | Myristoylated alanine-rich C-kinase substrate | 18 | 23 | 4 | 7 | 2.3 | 14 | 31.5 |
| Q5T2W1 | Na(+)/H(+) exchange regulatory cofactor NHE-RF3 | 44 | 25 | 9 | 14 | 3.8 | 65 | 57.1 |
| D6RHD2 | Protein sprouty homolog 1 (Fragment) | 14 | 17 | 3 | 4 | 2.0 | 7 | 22.9 |
| P00352 | Retinal dehydrogenase 1 | 250 | 54 | 33 | 76 | 2.5 | 62 | 54.8 |
| P50453 | Serpin B9 | 54 | 41 | 10 | 15 | 1.5 | 11 | 42.4 |
| O75368 | SH3 domain-binding glutamic acid-rich-like protein | 22 | 51 | 5 | 6 | 1.9 | 42 | 12.8 |

| C9JIM8 | Solute carrier family 2, facilitated glucose transporter member 1 (Fragment) | 8 | 8 | 2 | 2 | 1.7 | 29 | 27.2 |
| --- | --- | --- | --- | --- | --- | --- | --- | --- |
| P52788 | Spermine synthase | 50 | 42 | 10 | 14 | 1.6 | 19 | 41.2 |
| Q6ZVM7 | TOM1-like protein 2 | 149 | 50 | 18 | 40 | 1.5 | 25 | 55.5 |
| Q01995 | Transgelin | 329 | 89 | 26 | 100 | 1.5 | 27 | 22.6 |
| Q9BVC6 | Transmembrane protein 109 | 10 | 8 | 3 | 4 | 1.7 | 23 | 26.2 |
| C9J7Z4 | tRNA-splicing endonuclease subunit Sen2 (Fragment) | 10 | 6 | 2 | 3 | 1.5 | 25 | 43.1 |
| Q6BDS2 | UHRF1-binding protein 1 | 6 | 2 | 2 | 2 | 1.6 | 46 | 159.4 |
