# Supplementary material for: Downregulation of vimentin expression increased drug resistance in ovarian cancer cells
Source: Oncotarget. 2016 Jun 13;7(29):45876–88. doi: 10.18632/oncotarget.9970 (PMC5216767; doi:10.18632/oncotarget.9970)
Supplement: Supplementary file 5 [file oncotarget-07-45876-s005.docx]

| Accession | Description | Score | Coverage (%) | Unique Peptides | PSMs | VIM-KN/control | Ratio Variability [%] | MW  [kDa] |
| --- | --- | --- | --- | --- | --- | --- | --- | --- |
| Q08043 | Alpha-actinin-3 | 82 | 17 | 5 | 25 | 0.5 | 52 | 103.2 |
| Q16352 | Alpha-internexin | 378 | 69 | 40 | 132 | 0.4 | 84 | 55.4 |
| Q12797 | Aspartyl/asparaginyl beta-hydroxylase | 139 | 31 | 19 | 36 | 0.6 | 41 | 85.8 |
| A9UF05 | BCR/ABL fusion protein isoform Y3 | 9 | 7 | 2 | 3 | 0.6 | 69 | 51.4 |
| P61769 | Beta-2-microglobulin | 8 | 17 | 2 | 3 | 0.6 | 43 | 13.7 |
| Q9P2M7 | Cingulin | 11 | 7 | 7 | 7 | 0.6 | 29 | 136.3 |
| Q14019 | Coactosin-like protein | 88 | 93 | 17 | 31 | 0.6 | 47 | 15.9 |
| Q9Y281 | Cofilin-2 | 282 | 87 | 15 | 94 | 0.5 | 39 | 18.7 |
| P12277 | Creatine kinase B-type | 73 | 38 | 11 | 19 | 0.4 | 79 | 42.6 |
| P52943 | Cysteine-rich protein 2 | 51 | 53 | 9 | 14 | 0.6 | 44 | 22.5 |
| P15924 | Desmoplakin | 233 | 28 | 66 | 79 | 0.6 | 36 | 331.6 |
| B4DX66 | Epidermal growth factor receptor kinase substrate 8 | 10 | 9 | 4 | 4 | 0.6 | 19 | 62.8 |
| Q16658 | Fascin | 32 | 23 | 8 | 10 | 0.6 | 30 | 54.5 |
| O95864 | Fatty acid desaturase 2 | 9 | 7 | 3 | 3 | 0.5 | 23 | 52.2 |
| P09382 | Galectin-1 | 253 | 82 | 16 | 70 | 0.6 | 17 | 14.7 |
| F5H2U8 | High mobility group protein HMGI-C | 18 | 60 | 6 | 8 | 0.5 | 27 | 10.8 |
| P16402 | Histone H1.3 | 92 | 40 | 3 | 29 | 0.6 | 6 | 22.3 |
| Q8N257 | Histone H2B type 3-B | 470 | 72 | 2 | 184 | 0.5 | 13 | 13.9 |
| Q15475 | Homeobox protein SIX1 | 7 | 8 | 2 | 2 | 0.5 | 42 | 32.2 |
| Q14571 | Inositol 1,4,5-trisphosphate receptor type 2 | 11 | 1 | 2 | 4 | 0.6 | 15 | 307.9 |

| O00468-2 | Isoform 2 of Agrin | 36 | 6 | 9 | 11 | 0.6 | 56 | 205.4 |
| --- | --- | --- | --- | --- | --- | --- | --- | --- |
| Q99767-2 | Isoform 2 of Amyloid beta A4 precursor protein-binding family A member 2 | 6 | 5 | 2 | 2 | 0.6 | 40 | 81.1 |
| Q14004-2 | Isoform 2 of Cyclin-dependent kinase 13 | 8 | 2 | 3 | 4 | 0.5 | 18 | 158.3 |
| Q7Z5L9-2 | Isoform 2 of Interferon regulatory factor 2-binding protein 2 | 16 | 8 | 3 | 5 | 0.6 | 31 | 59.4 |
| Q27J81-2 | Isoform 2 of Inverted formin-2 | 20 | 9 | 6 | 6 | 0.5 | 54 | 134.5 |
| Q9NQ29-2 | Isoform 2 of Putative RNA-binding protein Luc7-like 1 | 75 | 32 | 4 | 20 | 0.6 | 19 | 38.4 |
| P02774-2 | Isoform 2 of Vitamin D-binding protein | 7 | 7 | 2 | 2 | 0.6 | 57 | 39.5 |
| Q86UU0-3 | Isoform 3 of B-cell CLL/lymphoma 9-like protein | 8 | 2 | 2 | 2 | 0.6 | 33 | 145.8 |
| Q14103-3 | Isoform 3 of Heterogeneous nuclear ribonucleoprotein D0 | 269 | 59 | 2 | 83 | 0.6 | 0 | 32.8 |
| A1L390-3 | Isoform 3 of Pleckstrin homology domain-containing family G member 3 | 9 | 4 | 4 | 4 | 0.6 | 22 | 128.2 |
| P84101-4 | Isoform 4 of Small EDRK-rich factor 2 | 15 | 80 | 6 | 7 | 0.6 | 14 | 5.2 |
| Q9UBC3-7 | Isoform 7 of DNA (cytosine-5)-methyltransferase 3B | 6 | 4 | 2 | 2 | 0.6 | 50 | 77.7 |
| Q15911-2 | Isoform B of Zinc finger homeobox protein 3 | 10 | 1 | 4 | 4 | 0.6 | 8 | 306.5 |

| Q14141-2 | Isoform I of Septin-6 | 31 | 12 | 3 | 11 | 0.6 | 18 | 48.8 |
| --- | --- | --- | --- | --- | --- | --- | --- | --- |
| P06213-2 | Isoform Short of Insulin receptor | 11 | 2 | 2 | 3 | 0.6 | 51 | 155.0 |
| B4DKL4 | Lipolysis-stimulated lipoprotein receptor | 20 | 19 | 6 | 6 | 0.5 | 30 | 54.5 |
| O75096 | Low-density lipoprotein receptor-related protein 4 | 6 | 2 | 3 | 3 | 0.6 | 8 | 211.9 |
| O14745 | Na(+)/H(+) exchange regulatory cofactor NHE-RF1 | 92 | 46 | 13 | 24 | 0.6 | 36 | 38.8 |
| P48681 | Nestin | 407 | 39 | 64 | 112 | 0.4 | 76 | 177.3 |
| Q09666 | Neuroblast differentiation-associated protein AHNAK | 1302 | 64 | 212 | 383 | 0.6 | 35 | 628.7 |
| F8VUJ3 | Protein POC1B-GALNT4 | 10 | 8 | 3 | 3 | 0.5 | 38 | 66.2 |
| H7C2G0 | SH3 domain-binding glutamic acid-rich protein (Fragment) | 8 | 22 | 2 | 3 | 0.6 | 82 | 15.3 |
| B7Z5G4 | Stathmin | 17 | 11 | 2 | 8 | 0.5 | 48 | 19.8 |
| Q99081 | Transcription factor 12 | 10 | 7 | 2 | 3 | 0.6 | 21 | 72.9 |
| M0R0H5 | Urokinase plasminogen activator surface receptor (Fragment) | 14 | 27 | 3 | 4 | 0.4 | 39 | 17.0 |
| B8ZZT4 | Vesicle-associated membrane protein 8 | 10 | 18 | 2 | 3 | 0.6 | 11 | 11.2 |
| P08670 | Vimentin | 8213 | 94 | 80 | 2807 | 0.5 | 50 | 53.6 |
| Q6P4I2 | WD repeat-containing protein 73 | 9 | 14 | 3 | 3 | 0.6 | 23 | 41.7 |
